# Scaling Effects of Elevation Data on Urban Nonpoint Source Pollution Simulations

**DOI:** 10.3390/e21010053

**Published:** 2019-01-11

**Authors:** Ying Dai, Lei Chen, Pu Zhang, Yuechen Xiao, Zhenyao Shen

**Affiliations:** State Key Laboratory of Water Environment Simulation, School of Environment, Beijing Normal University, Beijing 100875, China

**Keywords:** DEM data, scale effect, urban pollution, nonpoint source pollution, information entropy

## Abstract

The scale effects of digital elevation models (DEM) on hydrology and nonpoint source (NPS) pollution simulations have been widely reported for natural watersheds but seldom studied for urban catchments. In this study, the scale effect of DEM data on the rainfall-runoff and NPS pollution was studied in a typical urban catchment in China. Models were constructed based on the DEM data of nine different resolutions. The conventional model performance indicators and the information entropy method were applied together to evaluate the scale effects. Based on the results, scaling effects and a resolution threshold of DEM data exist for urban NPS pollution simulations. Compared with natural watersheds, the urban NPS pollution simulations were primarily affected by the local terrain due to the overall flat terrain and dense sewer inlet distribution. The overland process simulation responded more sensitively than the catchment outlet, showing prolonged times of concentration for impervious areas with decreasing DEM resolution. The diverse spatial distributions and accumulation magnitudes of pollutants could lead to different simulation responses to scaling effects. This paper provides information about the specific characteristics of the scale effects of DEM data in a typical urban catchment, and these results can be extrapolated to other similar catchments as a reference for data collection.

## 1. Introduction

Nonpoint source (NPS) pollution has become an important concern for urban water environment management as point source pollution has gradually been controlled [1]. As rainfall runoff-induced pollution, overland wash-off and the transmission process of pollutants are critical to the final pollution discharge [2,3]. Therefore, a good understanding of the overland NPS pollution process is helpful for planning pollution control measures.

Distributed urban NPS pollution models are common tools for studying the overland NPS pollution process as they possess physically-based routing algorithms and spatially heterogeneous structures [4]. For these models, high-resolution spatial input data, such as GIS maps for topography and geography features, and drainage data are generally needed to generate accurate simulations [5,6]. However, such detailed data are often difficult to obtain due to secrecy demands, absent records or the high costs of observation. If high-precision data are not available, coarse data are usually used as alternates, but they will consequently affect the simulation results. Specifically, coarse topographical and geographical data affect the overland routing process simulation by inaccurately identifying land surface features [7]. On the other hand, coarse drainage data may lead to the simplified replication of overland processes due to the coarse delineation of sub-catchments [8]. Therefore, an insight into the scale effect of spatial input data on model performance is necessary in order to determine the appropriate scale to balance the cost efficiency of data collection and the simulation accuracy.

Current related studies for urban catchments mainly pertain to the scale effects from land use data, drainage data or rain gauges. Meanwhile, few studies examine the topographical data, probably due to the relatively flat terrain in urban areas and the complex interference from human factors such as the drainage by sewer systems [9]. In addition, in widely used sub-catchment-based urban NPS pollution models, such as the storm water management model (SWMM) and the hydrological simulation program—FORTRAN (HSPF). The topological data, which is usually digitalized as the digital elevation model (DEM), mainly provides a reference for delineating sub-catchments and setting their average slope parameters, instead of being a direct model input [10,11]. Therefore, the identified terrain difference with different DEM resolutions may be easily averaged or neglected when the sub-catchment does not match the DEM raster. Through related studies in natural watersheds, a significant influence of the DEM resolution on the NPS pollution simulations has already been revealed [7,12]. However, it is inappropriate to extrapolate these results in natural watersheds directly to urban catchments, due to their different mechanisms of NPS pollution yielding and routing [13]. To provide a reference for data collection and the simulations in data-scarce urban areas, the impacts of DEM resolutions on urban NPS pollution still need to be explored. 

Additionally, evaluation indicators might be another barrier to studying the scaling effect. Traditionally, pair-wise comparison between predicted and observed data at the watershed outlet is a common method to evaluate the scale effect. For this, a few goodness-of-fit indicators are used, such as the Nash–Sutcliffe efficiency coefficient (NSE), coefficients of determination (R^2^), and the relative difference (RD) [7,14]. However, this point-to-point evaluation may lead to the loss of information that describes the spatial complexity and uncertainty of the NPS pollution processes. Although some qualitative analyses have been conducted for overland situations with different DEM resolutions by identifying the changes in sub-watershed delineations and critical source area (CSA) distributions [15,16], the quantitative evaluation method is still absent. Information entropy is a concept borrowed from thermodynamics, and is used as a parameter to characterize the state of disorder of a given system [17]. Since it was first proposed by Shannon [18], information entropy has been effectively applied in a wide spectrum of fields, including environmental and water resources [19,20]. Based on the basic theory, information entropy measures the amount of uncertainty and chaos by accounting for all different possible occurrences and their probability distribution [21]. Therefore, information entropy enables the description of the spatially stochastic nature of NPS pollution and its response to the topological variation. 

In this study, we focused on the scaling effect of DEM data on the simulation of urban NPS pollution. To track the detailed overland process, a grid-based distributed model developed by our previous study [22], the cellular automata-based hydrology and NPS pollution model (CA-HNPSM), was used instead of the sub-catchment-based semi-distributed models. The point-to-point indicators and the information entropy method were both used to evaluate the scaling effects of DEM data. A typical small urban catchment in Beijing, China, was selected as the study area. The study results are expected to provide references for collecting DEM data with appropriate resolutions in urban NPS pollution studies.

## 2. Materials and Methods 

### 2.1. Study Area and Data Collection

**Figure 1:**
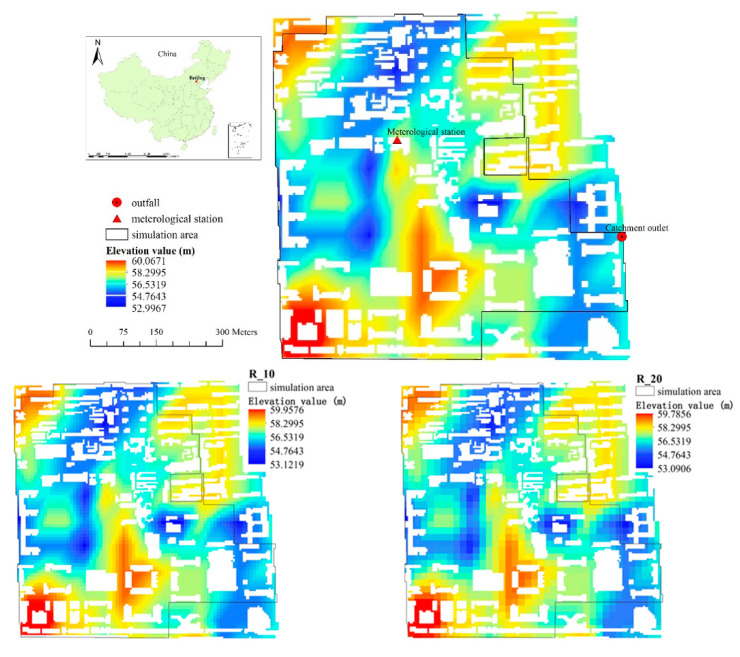

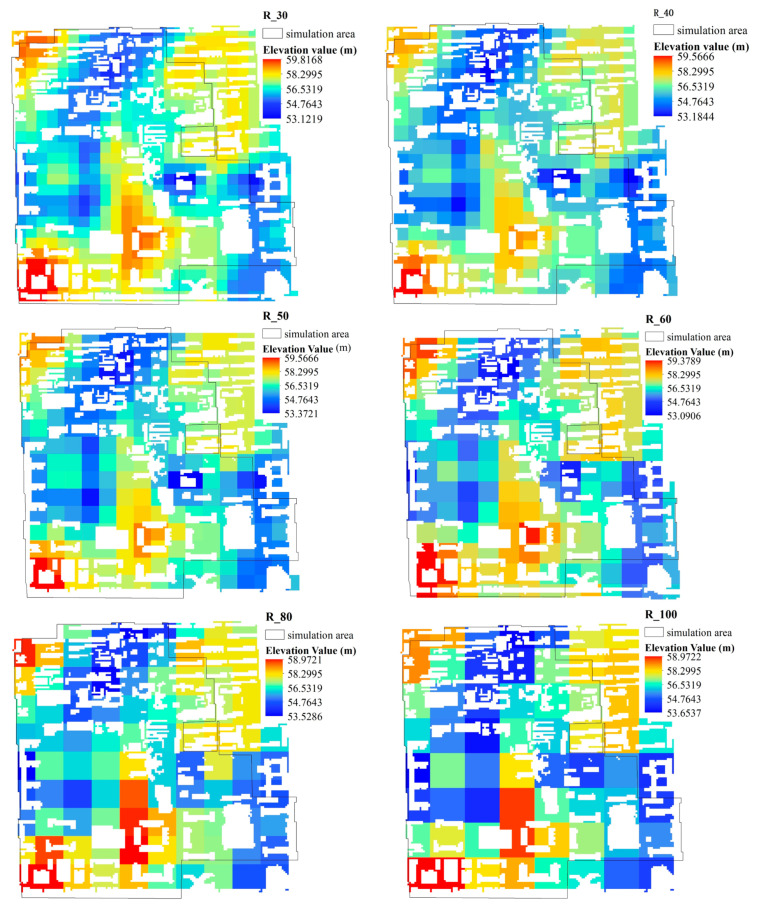
Land surface elevations of different resolutions.

**Figure 2:**
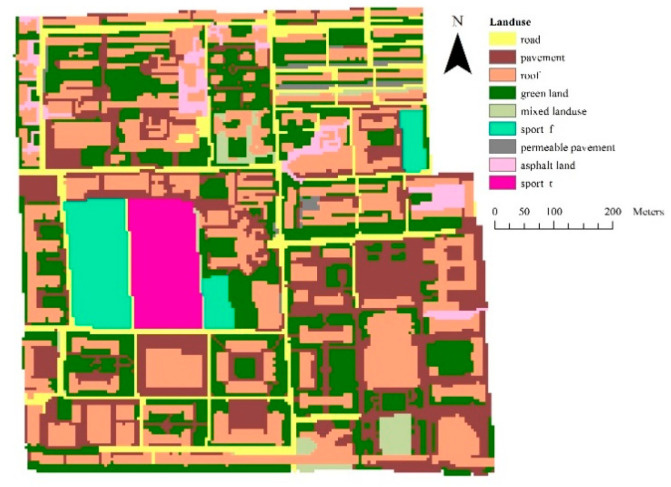
Land use and land cover types.

The study area is a catchment located on the campus of Beijing Normal University, and it covers a total area of 43.52 ha. Flat terrain dominates the topography of the area, and the land-surface elevation ranges from 52.99–60.07 m throughout the catchment (Figure 1). The study area consists of impervious land cover, including road, asphalt surfaces, concrete block pavements, building roofs and the sports fields laid with artificial turf, and pervious land covers (e.g., green spaces and permeable pavements). The impervious area makes up 65% of the whole catchment (Figure 2). The high proportion of impervious areas and the full coverage of the separate sewer system both make the study area a typical urban catchment. The study area belongs to a warm and semi-humid continental monsoon climate zone, and the rainy season generally lasts from June to August [23]. Among the rainfall events during the year, light rainfall accounts for the largest proportion, while heavy or torrential rainfall occurs less frequently, but contributes substantially to the total annual rainfall.

As for the input data, DEM data with a high resolution of 5 m, were collected from the Geographical Information Monitoring Cloud Platform and adjusted by 2670 observed elevation points altogether. Land cover data with a 5 m resolution, and drainage inlet locations were provided by the logistics department of the university. Rainfall was recorded at five-minute intervals during 2014 by a HOBO automated meteorological station (Onset, Bourne, MA, USA) on campus. To provide a reference for model calibration, hydrological and water quality monitoring was also conducted at the catchment outlet in 2014. The discharge flows were monitored at five-minute intervals from July to September 2014 by a current meter combined with a water level sensor (HACH FL900 by HACH, Loveland, Colorado, USA). The discharge samples were collected at different intervals considering the first flush effect during four independent rainfall events (Table 1). Indicators including chemical oxygen demand (COD), NH_4_-N and total phosphorus (TP) were used to test samples, using dichromate titration, salicylic acid spectrophotometry, and ammonium molybdate spectrophotometry, respectively. Due to the difficulty of manually collecting NPS pollution samples, the obtained observation data of NPS pollutants were limited. Therefore, the rainfall parameters, such as the total rainfall, rainfall intensity, rainfall pattern, and rainfall duration, were comprehensively considered when selecting representative rainfall events in order to reduce the uncertainties arising from a small sample group as far as possible. The four selected rainfall events basically cover the major rainfall characteristics in the study area. Specifically, rainfall-1 is a typical multi-peak long-lasting rainfall event with normal rainfall intensity but large total rainfall, while rainfall-2 is a typical short heavy rainfall with large rainfall intensity, rainfall-4 is a typical single-peak short light rainfall event, and rainfall-3 represents extreme rainfall conditions. 

### 2.2. Model Setup

#### 2.2.1. Model Description

A grid-based model, CA-HNPSM, was used for simulating the overland NPS pollution process instead of the sub-catchment-based methods of common urban NPS pollution models. The model treated the study area as a lattice space consisting of square cells. By considering the characteristic urban rainfall-runoff routing, the catchment was generalized into three layers, including a rooftop layer, land surface layer and a sewer pipe layer, which were connected by downspouts or sewer inlets. In each layer of rooftops and land surfaces, the rainfall-runoff and NPS pollution were routed according to water surface slopes calculated by the DEM input, coupled with runoff depths, while at the downspout located cells, the drainages were calculated and transferred from the roof layer to the land layer during each time step to form the hydrological connection between the two layers. This model was specifically described in our previous research [22]. Compared with other widely used urban NPS pollution models, the grid-based model delineates the study area into cells with the same size or smaller size compared to the raster resolution of the DEM data. It thus enables a full representation of the DEM data in the model, instead of averages for the sub-catchment. On this basis, a more synchronous response of the NPS pollution to the change in DEM resolution could be expected for a flat urban area. 

The calibration was conducted on an auto-calibration platform based on the combined algorithms of a non-dominated sorting genetic algorithm (NSGA-II) and multi-objective optimization [24]. Sets of parameters valued randomly within specific value ranges were brought into the model sequentially, and the corresponding simulation results at the catchment outlet were compared pair-wise for the observed discharge flows and pollution concentrations. The matching degrees of these comparisons were evaluated by the Nash-Sutcliffe efficiency coefficient (NSE) and the coefficient of determination ($R^{2}$), which were calculated as the following functions:

(1) $$\mathrm{NSE}=1-\frac{\sum_{i=1}^{n}{\left(O_{i}-P_{i}\right)}^{2}}{\sum_{i=1}^{n}{\left(O_{i}-\bar{O}\right)}^{2}}$$

(2) $$R^{2}={\left(\frac{Cov\left(O_{i},P_{i}\right)}{\sqrt{Var\left[O_{i}\right]Var\left[P_{i}\right]}}\right)}^{2}$$

where *O* is the observed value, *P* is the predicted value, n is the number of data points, and $\bar{O}$ is the mean observed values. The monitored discharge flow, COD, NH_4_-N, and TP concentrations of two rainfall events (rainfall 1 and 2) were used for the calibrations, and the other two rainfall events (rainfall 3 and 4) were used for the validations. Finally, a set of parameters for all the rainfall events were obtained.

#### 2.2.2. Setup of DEM Inputs

Models with different resolution levels were set up based on DEM data of 5 m, 10 m, 20 m, 30 m, 40 m, 50 m, 60 m, 80 m and 100 m raster size, and they were named as R-5, R-10, R-20, R-30, R-40, R-50, R-60, R-80, R-100, respectively. The coarser DEM inputs were obtained by resampling the 5 m × 5 m DEM using the nearest neighbour technique in the ArcGIS 10.2 software package. Despite the different resolutions of DEM data, the cell size of the model was still 5 m to maintain the correspondence with the resolution of the land cover input. Thus, the elevation of each cell was still guaranteed to be uniform. The model parameters were only calibrated for the R-5 model, considering that the higher-resolution input data could result in more accurate parameters. Then, these calibrated parameters were fixed in the other eight models to avoid any influence beyond the DEM resolution on the simulation results.

#### 2.2.3. Scaling Effect Evaluation Method

NSE and R^2^ were also used to indicate the performance of the models with coarser DEM data. By comparing the performance of the different models, the scale effect of DEM data could be evaluated in terms of the prediction results at the catchment outlet. In addition, the information entropy method was introduced to further quantify the scale effect in view of the overland NPS pollution process. Information entropy is a measurement of the uncertainty, disorder and diversity of a particular attribute [25]. Its value is always negatively correlated with the effective information provided by a given system, which means that a larger information entropy value indicates a more chaotic system state [26]. In this study, information entropy was used to quantify the change in the spatial distributions of NPS pollution, and it was calculated as the following equation [27]. By comparing it with the R-5 model, a larger change in information entropy (ΔH) indicates a more significant effect of the scale change on the overland routing simulation.

(3) $$H\left(x\right)=-\overset{n}{\underset{i=1}{\sum }}P\left(x_{i}\right)\ln P\left(x_{i}\right)$$

where H(x) is the information entropy; i denotes a spatial unit; $x_{i}$ denotes rainfall-runoff and NPS pollution outputs from i; $P\left(x_{i}\right)$ denotes the percentage of the runoff or pollution output of i, which represents the probability of spatial distributions; and n denotes the number of spatial units.

## 3. Results and Discussion

### 3.1. Scale Effects on Model Performance

**Figure 3:**
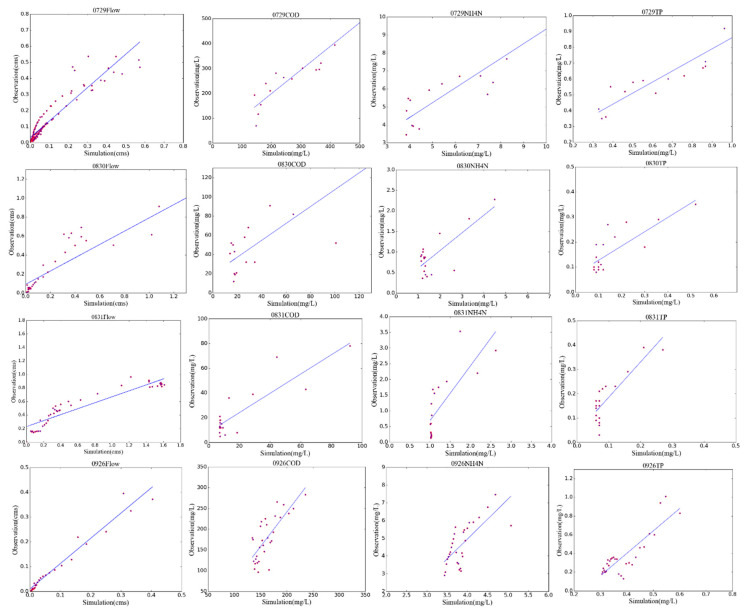
The scattergrams of runoff flows and pollutant concentrations.

First, the baseline model was calibrated and validated using the R-5 DEM data. The linear regressions of the simulations and observations of the R-5 model are given in Figure 3. The values of NSE and R^2^ for different rainfall events are listed in Table 2. For the rainfall-runoff simulation in the R-5 model, the NSE values exceeded 0.5 except for the 0831 rainfall, indicating that the hydrology simulation performance was at an acceptable level. For NPS pollution, the R-5 model also obtained acceptable performance, especially for COD, which indicated that the R-5 model, with the relatively higher-resolution input, could reflect the characteristics of NPS routing and discharge of this study area.

**Figure 4:**
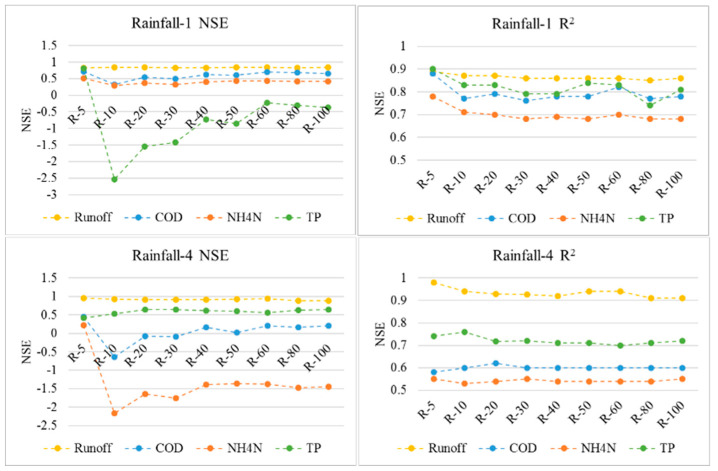
The model performance indicators.

By a comprehensive consideration of both the model performance and the diversity of rainfall conditions, two rainfall events (rainfall 1 and 4) were used to analyze the scale effects of DEM data on model performance. The NSE and R^2^ values for different scale levels were calculated. The results did not indicate an obvious scale effect on the hydrology simulations (Figure 4), which was probably due to the overall flat terrain in the urban catchment. The performance of the NPS pollution simulation was more clearly affected by the scale effect, as both the pollutant wash-off and transmission calculation doubled their dependence on the slopes derived from the DEM data [28]. As shown in Figure 4, the pollution simulation performance generally degraded as the DEM resolution decreased (in terms of the NSE values), except for the TP simulation in rainfall-4, while the R^2^ values still indicate satisfactory correlations between the predictions and observations. From the change trends of the NSE values, the most significant scale effects characterized by obvious NSE decreases occurred when the DEM resolution decreased from 5 m to 10 m. Then, the NSE values gradually recovered with the further scaling-up. After the resolution decreased to 40 m, the NSE values almost stabilized, indicating a threshold scale at which the low DEM resolutions seldom affected the model performance. However, this threshold scale has a contrast implication compared with some studies of natural watersheds, where the threshold scale is a resolution at which higher levels would not affect the model performance [7]. On this basis, the local terrain is inferred to be more important for the NPS pollution simulation in an overall flat urban catchment with dense sewer inlets. When the DEM resolution degrades to be too low to identify the local topographical changes, its scale effect correspondingly weakens.

### 3.2. Scale Effects on the Total Discharge Results

**Figure 5:**
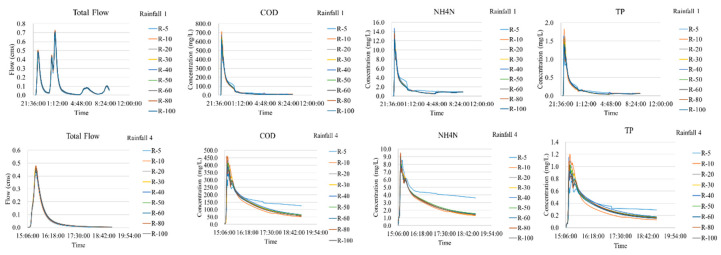
Predictions at the catchment outfall.

The prediction of the flows and pollution concentrations at the catchment outfall are shown in Figure 5. Generally, the predicted peak flows (the first peak flow in event No.1) and the rainfall-runoff ratios clearly increased when the DEM resolution changed from 5 m to 20 m, but they remained nearly stable or experienced only slight changes when the resolution decreased further. The responses of the predicted pollution concentrations were relatively more complicated, and they showed fluctuating trends in terms of the peak concentration (PC) and the event mean concentration (EMC). For the PC values, the scale effect was generally not conspicuous, especially when the resolution was below 10 m. For the EMC values, their response patterns differed in different rainfall events and were more sensitive in the heavier rainfall. 

### 3.3. Scale Effects on the Overland Routing Process

**Figure 6:**
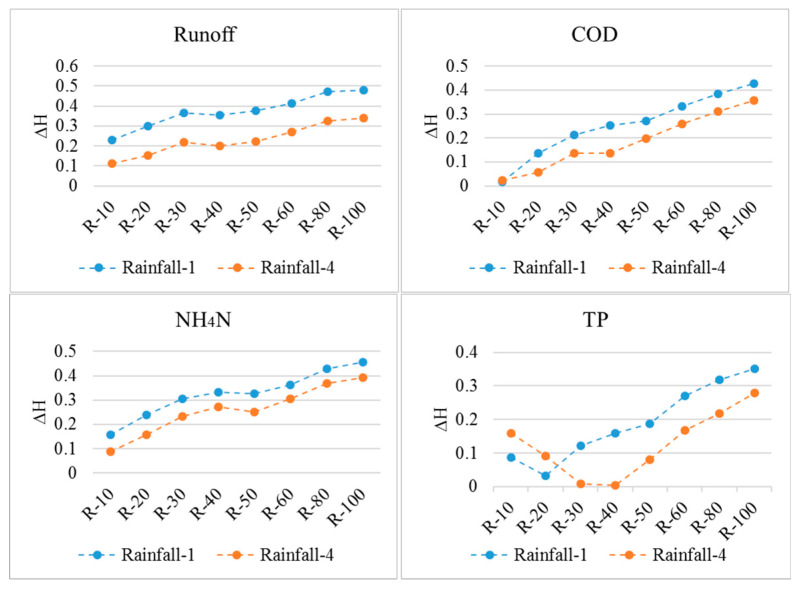
The absolute values of the relative changes in information entropy (ΔH).

**Figure 7:**
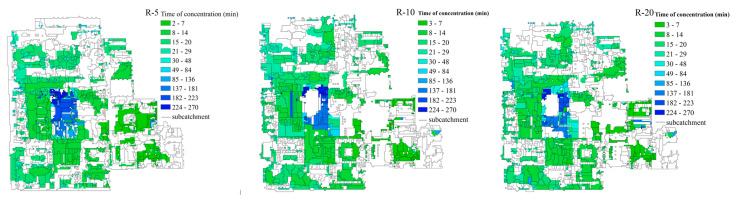

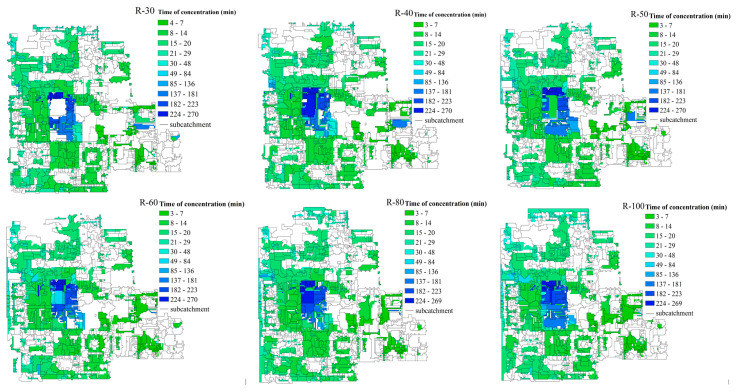
The sub-catchment delineation and the distribution of the concentration time.

**Figure 8:**
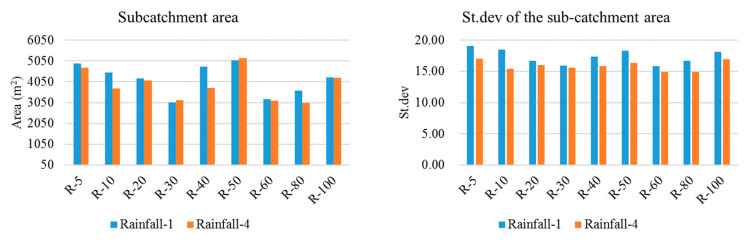
The change in sub-catchment delineations with the DEM resolution.

The ΔH was used to quantify the scale effects on overland rainfall-runoff and the NPS pollution distributions. The results (Figure 6) indicated that the scale effects on the overland hydrology simulations generally increased with the decrease in the DEM resolution, except for a slight drop at the resolution of 40 m. The resolution of 80 m could be regarded as the threshold for the scale effect on overland hydrology simulations, as the ΔH nearly stabilized below this resolution. According to the comparison between rainfall events, the scale effects on the overland hydrology process appeared to be also more significant in the heavier rainfall event (rainfall-1). The deeper overland runoff water in the heavier rainfall, which could exaggerate the effect of the slope change on the runoff rate calculation according to the Manning equation, may be the reason for the rainfall-induced difference in scale effect. Taking rainfall-1 as an example, Figure 7 shows the obvious change in the overland routing process in terms of the sub-catchment delineations and concentration time since the runoff generation to the runoff discharge at the outfall. Blank areas inside the sub-catchments in Figure 7 indicate the areas without hydrological connections to the sewer network, which would not contribute to the discharge at the outfall. By referring to the land cover distribution shown in Figure 2, a concentration time lower than 48 min was mainly distributed in impervious areas, while the longer concentration times occurred in pervious areas. With the decrease in DEM resolution, the total drainage area indicated by the nonzero concentration time tended to increase, and the concentration time was generally prolonged for impervious areas but shortened for pervious areas. Both indicated a more dispersive routing process due to the flatter terrain identified. According to the statistics, the sub-catchment area and the standard deviation (St.dev) of the sub-catchment areas both fluctuated with the DEM resolution (Figure 8). 

Regarding the ΔH values for overland NPS pollution, the variation trends of the ΔH values for COD and NH_4_N were similar to the hydrology simulation. This indicated a better correlation between the overland COD and NH_4_N pollution process and the hydrology process. Meanwhile, the ΔH values for TP had clear discrepancies. The differences in the scale effects between pollutants may be attributed to their diverse spatial distributions and the magnitudes of dry weather accumulations. Specifically, the COD mainly accumulated on impervious areas where the overland flow was fully associated with the overland NPS pollution process. Furthermore, the overland NPS pollution may depend more on the overland flow for pollutants with larger accumulation loads, while being limited by the loads for pollutants with less accumulation, which may explain the different scale effects for the NH_4_N and TP simulations.

Compared with the catchment outlet, the overland process responded more sensitively to the scale effect in a wider resolution range, as the change in slope directly affected the overland flow. When the DEM resolution degraded to the level of 40 m, the simulation tended to be stable regarding the model performance for predictions at the catchment outlet. Meanwhile, the overland flow continued to become more uncertain due to the flatter local terrain identified by the further decreasing DEM resolution. Simultaneously, the flatter terrain meant that the overland flow was driven more by the drainage through the sewer pipe inlets. Thus, the scale effect on the overland routing process tended to be limited by the spatial distribution density of the sewer inlets, and it stabilized when the resolution increased beyond most of the sub-catchment radius.

## 4. Conclusions

In this study, the scaling effect of DEM data on urban NPS pollution was quantified. Based on the results, scaling effects and the resolution threshold of DEM data exist for the urban NPS pollution simulations. Compared with natural watersheds, the urban NPS pollution simulations were primarily affected by the local terrain due to the overall flat terrain and dense sewer inlet distribution. The overland process simulation responded more sensitively than the catchment outlet and showed a prolonged concentration time for impervious areas with decreasing DEM resolution. The diverse spatial distribution and accumulation magnitude of pollutants led to different simulation responses to scaling effects. This paper analyzed the specific characteristics of the scaling effects of DEM data in a typical urban catchment, and these results can be extrapolated to other similar catchments as a reference for data collection.

However, the resolutions of multi-input data usually match each other, and their coupled scaling effects are also important concerns for practical NPS pollution simulations; they were not considered in this study and require further study. In addition, more measurements are suggested in the future to enrich the calibration data and reduce the prediction uncertainty.

## Figures and Tables

**Table 1:** Basic information on the monitored rainfall events.

Rainfall Event ID	Rainfall Date	Total Rainfall (mm)	Duration (min)	Peak Intensity (mm/h)	Rain Type
1	29 July 2014	35.7	400	43.28	Heavy rain
2	30 August 2014	29	105	69.6	Heavy rain
3	31 August 2014	70.76	165	86.2	Torrential rain
4	26 September 2014	7.8	20	50.4	Light rain

**Table 2:** The calibration and validation results indicated by NSE and R^2^.

	Event ID	Flow		COD		NH_4_-N		TP	
		NSE	R^2^	NSE	R^2^	NSE	R^2^	NSE	R^2^
Calibration	1	0.83	0.89	0.72	0.88	0.51	0.78	0.81	0.90
	2	0.77	0.85	0.62	0.85	−1.59	0.52	0.53	0.69
Validation	3	0.044	0.87	0.62	0.78	0.4	0.71	0.023	0.76
	4	0.95	0.98	0.45	0.58	0.22	0.55	0.42	0.74
